# Morphometric Analysis of Foramina in the Middle Cranial Fossa of Dogs: A Retrospective Cone-Beam CT Study

**DOI:** 10.3390/ani16121819

**Published:** 2026-06-12

**Authors:** Nimet Turgut, Sadullah Bahar, Tutku Mecit, Yağmur Çaltıner, Abdullah Bilal Çil

**Affiliations:** 1 Faculty of Veterinary Medicine, Department of Anatomy, Selcuk University, Konya 42130, Türkiye; sbahar@selcuk.edu.tr; 2 Institute of Health Sciences, Department of Veterinary Anatomy, Selcuk University, Konya 42130, Türkiye; 3 Faculty of Veterinary Medicine, Selcuk University, Konya 42130, Türkiye

**Keywords:** canidae, computed tomography, measurements, skull base, neurovascular structures

## Abstract

Although extensively studied in humans, data on the middle cranial fossa foramina remain limited in dogs, despite their different skull morphology and high relevance to veterinary neurology, surgery and oncology. In this retrospective anatomic study, we aimed to fill this gap by presenting the morphometric data of these foramina in domestic dogs of different breeds, ages, body weights, and skull sizes. The study used CBCT images of 40 dogs. Dogs were divided into three groups (small, medium, and large), regardless of sex, body weight, and breed, using neurocranium length. Then, morphological and morphometric analyses of the foramina were performed. The neurocranium length of each group differed significantly from the others (*p* < 0.001). In each group, the orbital fissure and round and oval foramina were bilaterally located rostrally to caudally and were of similar size (*p* > 0.05). While the orbital fissure was a canal in 80% of dogs, in dogs with medium and large skull sizes (17.5%), the spinous foramen showed variation, becoming both a foramen and a canal. The opening sizes increased along with the skull size (*p* < 0.001); the widest opening was the orbital fissure, and the narrowest opening (except for the spinous foramen) was the oval foramen. The findings may guide skull base surgeries, regional anesthesia, and the diagnosis of cranial nerve dysfunctions. Furthermore, a classification based on neurocranial length is anticipated to provide more objective craniometric measurements in animals with diverse head types and body weights.

## Simple Summary

In dogs with different skull types, the structural characteristics of the foramina, which serve as pathways for critical vessels and nerves, are important for the diagnosis of neurological diseases and surgical interventions. However, how the foramina are affected as skull size changes in dogs has not yet been fully elucidated. For this study, we used CT images of 40 healthy dogs (aged 1–17 years). Dogs were classified into three groups (small, medium, and large). In this grouping, neurocranium length, which successfully discriminated dog morphology, was utilized instead of the traditional skull index. In each group of dogs, the location and size of the foramina (orbital fissure, round and oval foramina) in the right and left halves of the skull were similar. In contrast, the presence of the foramen spinosum was not detected in dogs with a small skull morphology. Regardless of the breed and age of the dogs, although skull size and body weight increased, the cross-sectional shape on CT images of the foramina remained the same, but their size increased. These findings provide useful reference values for domestic dogs with different skull types and improve our understanding of the morphological and dimensional structure of the foramina.

## 1. Introduction

The middle cranial fossa is a morphologically complex intracranial region bounded at its base by the sphenoid bone and contains essential cranial nerves and vascular structures. This fossa contains bilateral openings, which have different names in different animal species owing to structural differences that allow the passage of neurovascular structures [1]. In dogs, the middle cranial fossa contains the orbital fissure (fissura orbitalis; ORF), the round foramen (foramen rotundum; RF), the oval foramen (foramen ovale; OF), and sometimes the spinous foramen (foramen spinosum; SF) [2,3]. The cranial fossa is not directly or easily accessible from the exterior.

Cone beam computed tomography (CBCT) scanners are systems with an X-ray tube providing a conical beam and flat panel detectors placed opposite each other, capable of making a partial or full rotation around a fixed patient. During rotation, 150–600 or more consecutive planar projection images can be captured from the field of view. These images are reconstructed, converted first into a three-dimensional (3D) dataset, and subsequently into cross-sectional images. These scanners are manufactured in different structures to serve various areas of use, such as C-arm, O-arm, and closed systems like conventional computed tomography (CT) [4,5,6,7,8]. Although CBCT scanners have limited success, particularly in soft tissue contrasting, they are preferred by veterinary clinics for many reasons, such as their ability to generate much thinner slice (0.08 mm) images compared to fan beam computed tomography (FBCT) scanners, allowing multimodal use such as digital radiography, panoramic imaging, and fluoroscopy, having low acquisition and operating costs, and being portable. With CBCT devices being specially manufactured and put into use for veterinary medicine, they have become much more accessible for animal patients today [4,9,10]. CBCT was initially used to diagnose animals and plan treatment. However, these imaging methods have provided new perspectives on animal anatomy.

With the process of domestication and the influence of intensive artificial selection applied, modern dog breeds exhibit a quite wide range of craniofacial morphological variation [11,12]. Additionally, in the diagnosis of cranial nerve dysfunctions and in oncological diagnostic and treatment applications in dogs, knowing the normal anatomical borders, dimensional data, and potential variations of the cranial fossa openings is of utmost importance [13,14,15,16,17]. However, current knowledge regarding the general anatomical structure of the foramina and canals located in the middle cranial fossa of dogs has been largely prepared and presented in anatomy textbooks using dry skulls. Therefore, there is a need for up-to-date topographic and morphometric information on these foramina at the skull base in dogs with different skull types, obtained through modern imaging methods. The purpose of this retrospective anatomic study is to describe the sectional and reconstructive morphology of the foramina located in the middle cranial fossa of dogs via CBCT images and to establish the initial preliminary reference data regarding their morphometric characteristics. The research results may assist clinicians and radiologists in the diagnosis of complex cranial syndromes due to compression at the skull base, such as bilateral cavernous sinus syndrome, oculomotor and trigeminal neuropathy, in the safe application of maxillary and mandibular nerve blocks in maxillofacial surgery, in transcranial or transoral surgical approaches for skull base tumors, and in stereotactic radiosurgery planning.

## 2. Materials and Methods

### 2.1. Animals and CBCT Data Sources

To conduct this retrospective anatomic study, we compiled 94 dog CBCT data from the radiology archives of two veterinary hospitals (See Acknowledgments), both of which used the same imaging modality (Vimago CT-CBCT, Epica Medical Innovations, San Clemente, CA, USA, scanning parameters; kVA: 80–90, mAs: 35–55, pixel size: 0.2 × 0.2 mm^2^ and 0.3 × 0.3 mm^2^, slice thickness: 0.2–0.3 mm). The data were transferred to DICOM program (RadiAnt DICOM Viewer (ver. 2025.1, Medixant, Poznan, Poland). https://www.radiantviewer.com/ (accessed on 5 December 2025)) by an anatomist (S.B.) with 17 years of experience in the field of radiology. The asymmetric development, anomaly, trauma, infection, tumor, image quality, and the bone and tooth structures of the head region were examined using the multiplanar reconstruction (MPR) and the 3D modules of program (RadiAnt DICOM Viewer, ver. 2025.1, Medixant, Poznan, Poland). Following the preliminary evaluation, a dataset of 40 dogs (20 females and 20 males, 1–17 years, 2.5–45 kg) was included in the study (Table S1, Figure S1). Cranial CBCT data comprised DICOM images collected from December 2021 to May 2024. The dogs belonged to 19 different breeds, but five dogs were of unidentified breeds (Table S1). Among these dogs, symphyseal separation of the mandible was found in one individual, a unilateral mandibular corpus fracture in another individual, alveolar destruction consistent with periodontitis in a third individual, and a compression fracture of the facial skeleton in a fourth individual. All procedures were performed after obtaining informed consent from the owners, approval from the hospital chief physician, and permission from the Selçuk University Faculty of Veterinary Medicine Experimental Animal Production and Research Center Ethics Committee (approval number: 2023/150).

### 2.2. Methods

All the involved researchers worked together to evaluate the cross-sectional and reconstructive anatomy of the middle cranial fossa before the analyses to minimize measurement-related errors and ensure coordination among researchers. A desktop computer with high processing power and high screen resolution (4K-29 inch) was used to evaluate and analyze the data. The data were evaluated at constant contrast and brightness levels within the bone window setting under low light conditions. The analysis was conducted independently by three investigators (N.T., S.B., and T.M.). The arithmetic mean of morphometric measurements was determined. The measurement values were defined in millimeters (mm) and presented as the mean ± standard deviation (SD). In CBCT sectional images of the middle cranial fossa, anatomical structures and linear measurements were labeled in the JPEG format using an image processing program (Adobe Photoshop 2025-ver. 27.3.0, Adobe system, San Jose, CA, USA). The anatomical datasets derived from DICOM images were subjected to segmentation via 3D modeling software (3D Slicer, Open source. https://www.slicer.org/ (accessed on 13 December 2025)) to create high-fidelity 3D digital reconstructions. We used classical anatomy sources [2,3] and Nomina Anatomica Veterinaria [18] to annotate anatomical structures using English terminology.

#### 2.2.1. Descriptive Craniometric Measurements and Grouping of Dogs

The dataset of each animal was imported into the DICOM imaging software, and the MPR module was activated. In this module, the head of the animal was oriented in the anatomical position by aligning the transverse plane parallel to the hard palate and the sagittal plane along the median plane. Using the linear measurement tool of the software, the following craniometric parameters were recorded: five lengths (skull length: SL, basal length: BL, viscerocranium length: VL, cranial length: CL, and neurocranium length: NL) in the sagittal CBCT section, and two widths (skull width: SW and neurocranium width: NW) in the dorsal and transverse CBCT section (Figure S2). Two distinct indices, the Skull Index (SI) and the Cranial Index (CI), were derived from these measurements using the following equations: SI = SW × 100/SL and CI = NW × 100/CL [3,19].

As the breed and body weight showed significant variations within the study population, it was challenging to establish definitive preliminary reference data for the middle cranial fossa openings. To address this problem, the dogs were grouped based on descriptive craniometric parameters to ensure that the analysis was more standardized. Consequently, all datasets were processed without differentiating between the sexes, and the dogs were classified into three distinct groups using NL as the primary reference. These are as follows: small breed (group 1; six females and four males, NL: 60.50 ± 2.81 mm), medium breed (group 2; five females and nine males, NL: 78.32 ± 7.03 mm), and large breed (group 3; nine females and seven males, NL: 111.89 ± 8.57 mm) (Table 1 and Table S1, Figure S1).

#### 2.2.2. Morphological and Morphometric Examinations of Foramina in the Middle Cranial Fossa

First, the dataset of each animal was imported into the MPR module of the DICOM imaging program. Then, the morphological features (including presence, location, shape, symmetry, and number) of the ORF, RF, OF, SF, and adjacent anatomical structures located in the middle cranial fossa were evaluated by moving in the transverse and dorsal CBCT sections of the image. The 3D middle cranial fossa models prepared in 3D, using a module of the same program, were also simultaneously included in these examinations. Only the morphological features of the accessory openings in the middle cranial fossa, which were not measurable, were determined.

**Figure 1:**
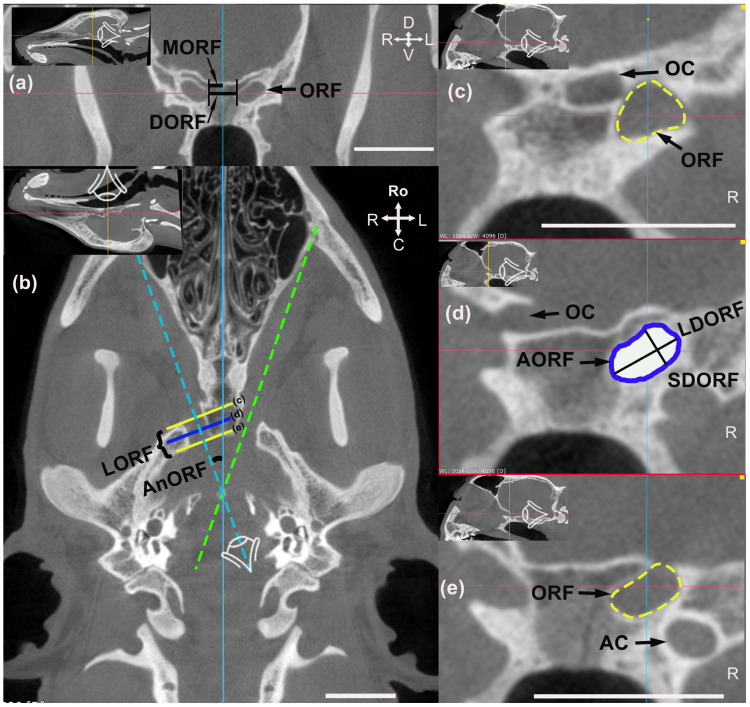
Orbital fissure and its morphometric measurements (right orbital fissure) in multiplanar CBCT images in male dog (Pointer) (No:36, group 3, 1 year ♂). (**a**) Transverse CBCT section [level between the rostral clinoid process and the rostral border of the chiasmatic sulcus], (**b**) dorsal CBCT section, (**c**) sagittal oblique CBCT section [extracranial opening of ORF], (**d**) sagittal oblique CBCT section [middle section of ORF-diameter and area measurements of the ORF were performed in this cross-sectional image], (**e**) sagittal oblique CBCT section [intracranial opening of the ORF]. AC, alar canal; AnORF, angle of orbital fissure; AORF, cross-sectional area of orbital fissure; C, caudal; DORF, distance between right and left orbital fissure; D, dorsal; L, left; LDORF, longer diameter of orbital fissure; LORF, length of canal of orbital fissure; MORF, distances from orbital fissure to midline; OC, optic canal; ORF, orbital fissure; R, right; Ro, rostral; SDORF, shorter diameter of orbital fissure; V, ventral. The images are taken from the multiplanar reconstruction (MPR) module of the DICOM software program. The thin red, yellow and blue lines shown in the images represent the reference axes (or lines) of the transverse, sagittal and dorsal (coronal) CBCT section, respectively. The images in panels (**c**,**d**), and e are magnified sections of the extracranial opening, middle part, and intracranial opening, respectively, of the canal through which the dashed blue line passes in b. Yellow dashed lines indicate the intracranial and extracranial openings of the ORF. Blue and green dashed lines are used to represent angle measurements. The perspectives belonging to CBCT are shown using the eye figure. All scale bars are 20 mm.

**Figure 2:**
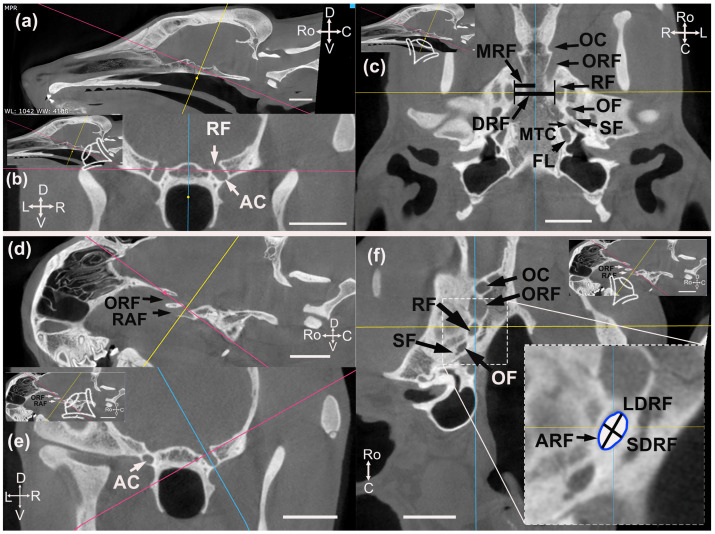
Round foramen and its morphometric measurements in multiplanar CBCT images in male dog (Pointer) (No: 36, group 3, 1 year ♂). (**a**) Sagittal CBCT section, (**b**) transverse CBCT section [the hypophyseal fossa level], (**c**) dorsal CBCT section, (**d**) sagittal oblique CBCT section [in this image, two perpendicular planes passing through the RF were inserted. Then, transverse oblique (**e**) and dorsal oblique (**f**) CBCT section images were created], (**e**) transverse oblique CBCT section, (**f**) dorsal oblique CBCT section. ARF, cross-sectional area of round foramen; DRF, distance between right and left round foramen; FL, foramen lacerum; LDRF, longer diameter of round foramen; MRF, distances from round foramen to midline; MTC, musculotubal canal; OF, oval foramen; ORF, orbital fissure; RAF, rostral alar foramen; RF, round foramen; SDRF, shorter diameter of round foramen; SF, spinous foramen. The inset image in the bottom right corner of panel (**f**) is a magnified view of the area demarcated by the dashed lines. See legend of Figure 1 for other abbreviations and explanations.

**Figure 3:**
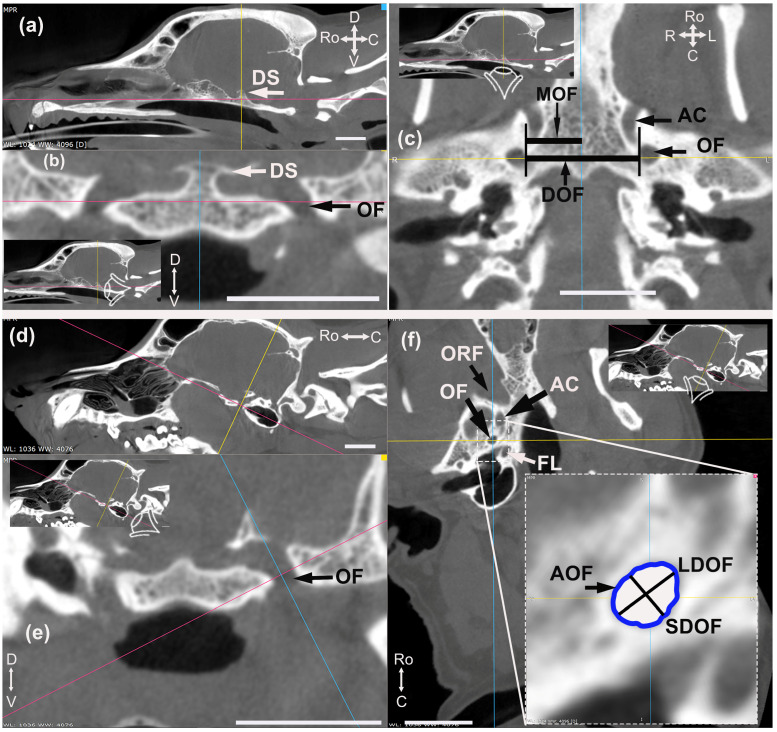
Oval foramen and its morphometric measurements in multiplanar CBCT images in male dog (Golden retriever) (No:22, group 2, 8 years ♂). (**a**) Sagittal CBCT section, (**b**) transverse CBCT section [the temporomandibular joint level], (**c**) dorsal CBCT section, (**d**) sagittal oblique CBCT section, (**e**) transverse oblique CBCT section (the dorsum sellae level), (**f**) dorsal oblique CBCT section. AOF, cross-sectional area of oval foramen; DOF, distance between right and left oval foramen; DS, dorsum sellae; LDOF, longer diameter of oval foramen; MOF, distance from OF to midline; OF, oval foramen; SDOF, shorter diameter of oval foramen. See legend of Figure 1 and Figure 2 for other abbreviations and explanations.

The diameter, area, and length of the foramina were measured on the MPR module in a transverse CBCT section (double oblique) and at high magnification. First, the positions of the foramina were determined in a transverse CBCT section (Figure 1a, Figure 2b and Figure 3b). Next, two planes were positioned parallel to the opening walls and passing through the center of the foramen (Figure 2d,e and Figure 3d,e). In a third plane, perpendicular to these planes, sectional views were created where the foramen was surrounded by bone 360°. By moving in this plane, two perpendicular diameters and area measurements with the greatest length were considered at the narrowest section of the foramen (Figure 2f and Figure 3f) [20,21,22]. The dorsal (or transverse) CBCT section was used to measure the distances between the medial wall of each foramen and the median plane (Figure 1a, Figure 2c and Figure 3c). In the dorsal CBCT section, the angle between the ORF and the median plane was measured (Figure 1b). The foramina were described as canal-like if the distance between the extracranial and intracranial opening exceeded 2 mm [23]. The extracranial and intracranial opening boundaries of the canal were determined, and the distance between these two openings was measured as the canal length (Figure 1b). The diameter and area of the narrowest part (approximately the middle one-third) of the canal were determined (Figure 1d) [22,24].

### 2.3. Statistical Analysis

All statistical analyses were performed using SPSS (version 31.0, IBM Corp., Armonk, NY, USA). Descriptive statistics are presented for categorical and continuous variables. The Shapiro–Wilk test was performed to determine whether the data followed a normal distribution. Levene’s test was performed to examine homogeneity of variance. Dependent variables (bilateral data) were compared by conducting paired sample *t*-tests, and one-way ANOVA was conducted to evaluate the mean differences among the three categorized groups. When ANOVA showed significant differences, Tukey’s post-hoc test was conducted to identify the source of the variance. Relationships between continuous variables were analyzed using the Pearson correlation coefficient. All results were considered to be statistically significant at *p* < 0.05, *p* < 0.01, and *p* < 0.001. To assess the inter-observer reliability among the three researchers, the intraclass correlation coefficient (ICC) was calculated with 95% confidence intervals. While an ICC above 0.75 is considered to be ideal, an ICC between 0 and 1 is acceptable [25,26].

## 3. Results

### 3.1. Craniometric Findings

The demographic data and craniometric measurement results of the dogs used in the study are summarized in Table 1. By analyzing the morphometric data, we found that the lowest and highest values for the parameters SL, BL, SW, VL, CL, NL, and NW were in groups 1 and 3, respectively (*p* < 0.001) (Table 1). The SI values between groups 1 and 2 were not statistically significant (*p* > 0.05). However, the differences in the SI values between group 3 and both group 1 (*p* = 0.002) and group 2 (*p* = 0.016) were significant. The CI values were significantly different among all canine groups (*p* < 0.001) (Table 1). The results of the correlation analysis (n = 40) demonstrated a strong correlation between SI and CI (*p* < 0.001). Similarly, a near-perfect positive correlation was found between NL and CL (*p* < 0.001). In contrast, moderate negative correlations were found between SI and the length parameters (NL and CL) (*p* < 0.001). Additionally, very strong negative correlations were found between CI and these lengths (NL and CL) (*p* < 0.001; Table S5).

### 3.2. Morphology Findings of Foramina in the Middle Cranial Fossa

**Figure 4:**
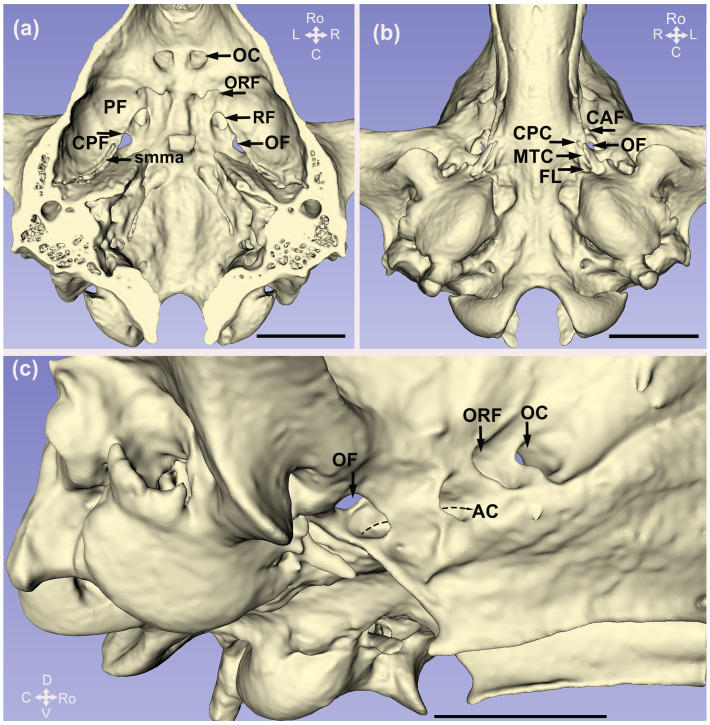
Dorsal (**a**), ventral (**b**), and lateral (**c**) views of the foraminaand canals located in the middle cranial fossa in the 3D skull model of female dog (Golden retriever) (No:29, group 3, 7 years ♀). AC, alar canal; C, caudal; CAF, caudal alar foramen; CPC, caudal opening of pterygoid canal; CPF, crista of the piriform fossa; D, dorsal; FL, foramen lacerum; MTC, musculotubal canal; OC, optic canal; OF, oval foramen; ORF, orbital fissure; PF, piriform fossa; Ro, rostral; RF, round foramen; smma, sulcus of middle meningeal artery; V, ventral. 3D images were prepared using modeling program. All scale bars are 20 mm.

**Figure 5:**
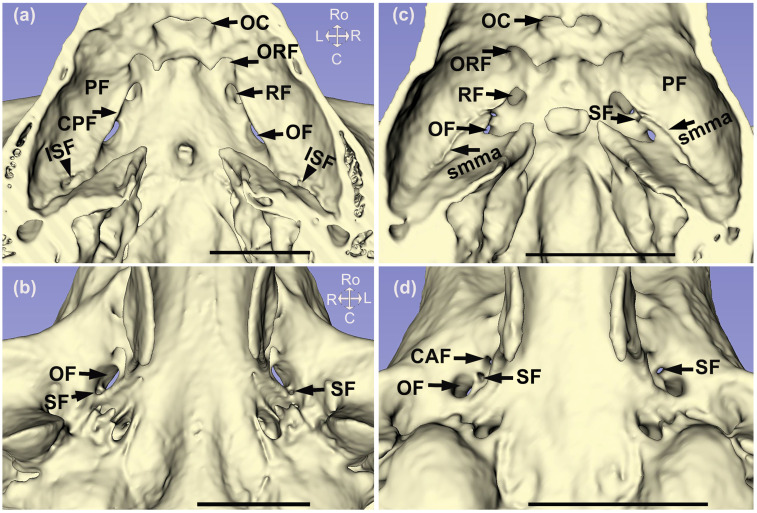
Dorsal and ventral views of different formations of the spinous foramen in 3D skull models of male dogs (Pointer and Terrier) ((**a**,**b**) = No:36, group 3, 1 year ♂; (**c**,**d**) = No:15, group 3, 1 year ♂) [caudal canal formations (**a**,**b**) were observed in five animals, while rostral foramen formations (**c**,**d**) were observed in two animals]. CPF, crista of the piriform fossa; ISF, internal opening of spinous foramen. See legend of Figure 4 for other abbreviations and explanations.

We analyzed 40 canine head CBCT datasets (1025.12 ±321.80). The CBCT images revealed that although their shapes and sizes varied across all three groups of dogs, the orbital fissure (ORF), round foramen (RF), and oval foramen (OF) were located bilaterally in all animals. These three openings were situated between the hypophyseal fossa, which is in the midline, and the piriform fossa on either side (Figure 4a and Figure 5a,c).

The ORFs were observed as symmetrical oval openings, diverging from the caudal to the rostral direction, in transverse CBCT sections passing between the rostral clinoid process and the rostral border of the chiasmatic sulcus (Figure 1a). The intra–extracranial openings were wide (Figure 1c,e), narrow in the middle section (Figure 1d), and formed an oblique oval canal along its course. The RFs, in the transverse sections passing through the level of the hypophyseal fossa, appeared as symmetrically positioned openings that led into the dorsal wall of the alar canal (Figure 2c). The RF had an oval shape and an orientation rostrolateral. The OFs, at the level of the temporomandibular joint, were symmetric in transverse sections located between the caudal border of the hypophyseal fossa and the caudal border of the dorsum sellae (Figure 3b,c). The OF appeared as an oval canal shape in three dogs within group 3 (Female: 2, Male: 1). In the remaining dogs (n = 37), the structure appeared as a foramen. The spinous foramina (SFs) were present bilaterally in seven of the dogs (17.5%). In five dogs, the SFwas located caudolaterally to the OF (group 2: n = 2; group 3: n = 3) (Figure 2f and Figure 5b), and in two dogs, it was located rostrally to the OF (group 2: n = 1; group 3: n = 1). The caudolateral SF (one female and four males) appeared as bilateral canals oriented in the caudodorsal direction within the wing of the basisphenoid bone (Figure 5a,b). The extracranial opening of the canal was circular (diameter: 0.79 ± 0.05 mm) and located immediately caudolateral to the OF (Figure 5b). The intracranial opening was located at the sphenopetrosal fissure (Figure 5a). The rostral SF was situated between the OF and RF (Figure 5c,d) and was wider on the left side (left side: 1.21 mm; right side: 0.72 mm).

### 3.3. Morphometric Findings of Foramina in the Middle Cranial Fossa

The agreement between diameter, area, length, and angle measurements for 240 openings, performed by three independent researchers, was statistically significant (*p* < 0.001). Except for four parameters, the ICC values for other parameters exceeded the 0.90 threshold, indicating ‘excellent agreement’ (Table S2). These morphometric measurements, performed using CBCT images in dogs for the first time, showed high reproducibility. The morphometric measurements of the right and left openings located in the middle cranial fossa of the three groups of dogs are presented in Table 2. The correlations between the opening parameters and age, weight, and craniometric data are provided group-wise in Table S3, while the results obtained by pooling all groups are presented in Table S4.

Except for the MRF value in group 2 (*p* > 0.05), the right and left ORF, RF, and OF dimensions within each group were similar. Based on the morphometric data obtained, it was determined that the ORF was the largest opening in the middle cranial fossa, and the OF was the second smallest opening, following the SF. Except for the index and angle, the ORF, RF, and OF parameters were highest in group 3 dogs and lowest in group 1 dogs (*p* < 0.001; Table 2). A significant positive correlation was found between the ORF, RF, and OF morphometric parameters and both body weight and craniometric measurements across all groups (*p* < 0.05; Tables S3 and S4).

## 4. Discussion

This study provides a comprehensive description of the cross-sectional radiological and reconstructive anatomical structure and morphometric features of the openings located in the middle cranial fossa of dogs.

### 4.1. Craniometric Parameters and Animal Classification

Domestic dogs are traditionally classified as brachycephalic, mesocephalic, and dolichocephalic according to the skull index (SI) [3,27,28,29]. When the 40 dogs examined in our study were classified according to the SI ranges reported by Sokołowski et al. [27,28], they were categorized as 2 dolichocephalic, 32 mesocephalic, and 6 brachycephalic. In contrast, when the SI threshold values of Ichikawa et al. [29] were taken as a basis, the same animals were classified as 3 dolichocephalic, 15 mesocephalic, and 22 brachycephalic. Such high variability in the index threshold values in the literature [3,27,28,29] demonstrates that SI-based classification does not provide a precise and standard differentiation, especially in dogs with different body weights and age ranges. The neurocranium and viscerocranium parts of the skull possess different growth characteristics. It is known that the neurocranium largely completes its development in the early postnatal period and exhibits a more stable morphology [1,2,3]. To overcome this methodological uncertainty, a classification approach based on linear measurements of the neurocranium was adopted. As a result of the analysis performed based on neurocranium length (NL), the animals in the study were divided into three distinct groups (small, medium, and large dogs) (*p* < 0.001; Table 1 and Table S1, Figure S1). This classification allowed for a clearer analysis of the morphological and morphometric characteristics of the foramina in the middle cranial fossa and their relationships with skull size in animals with different body sizes and age ranges.

### 4.2. Morphological Characteristics and Variations of Foramina

In ruminants and pigs, the RF is fused with the ORF [1,2]. In Equidae, the opening is located ventrolateral to the ORF and joins the alar canal rostrally [2]. In dogs, the opening is round and shallow and located caudally and slightly laterally to the ORF, entering the alar canal from the cranial cavity [2,3]. In our study, the RF (Figure 2b) appeared as a narrow, oval-shaped, and symmetrical structure in transverse sections passing through the hypophyseal fossa (Figure 2d). The statistical similarity of the RF index among the examined dog groups indicated that although the cranial size increased, the geometric form of the aperture was preserved (Table 2).

In horses and swine, the OF is described as an incisura located lateral to the foramen lacerum [2]. In cattle (85.7%), it is identified in transverse sections at the level of the dorsum sellae as a canal, with a wide oval shape [22]. The OF is a large opening in dogs; it opens directly into the cranial cavity 5 mm medial to the temporomandibular joint [3]. As a result of the current study, the OF (Figure 3b) was symmetric in transverse sections between the hypophyseal fossa and the dorsum sellae (Figure 3b) in dogs. In 92.5% of the dogs, the OF was a narrow, oval-shaped, and straight-walled foramen, whereas 7.5% exhibited an oval-shaped canal. The OF on the right side in group 1 dogs was more transversely compressed and narrower than the OF of the other groups of dogs (Table 2). This suggests that the right OF index may serve as a more sensitive and distinctive marker for differentiating between dog groups.

### 4.3. Morphometric Evaluation of the Foramina

A distance exceeding the 2 mm threshold between the intra–extracranial openings is accepted as a criterion for defining the related structure as ‘canal-like’ from a morphological perspective [23]. Length of the canal of the ORF was the greatest in group 3 and the least in group 1 (*p* < 0.001, Table 2). Analyses have shown that there might be a direct relationship between skull size and the anatomical structure of the opening. While the ORF had a complete canal form in all specimens of group 3, this rate decreased to 92.9% (13/14) in group 2 and decreased significantly to 30% (3/10) in group 1. These findings indicate that parallel to the reduction in skull size, the ORF preserves a foramen morphology rather than a canal form.

The OF diameter is around 2.7 × 1.6 mm in New Zealand rabbits [30]; 11.2 × 7.3 mm on the right and 11.3 × 7.5 mm on the left in Holstein cattle [22]; and between 1.08 and 6.50 mm on the right and 1.18 and 6.88 mm on the left across eight different primate species [31]. In our study, intra-group evaluations confirm the presence of bilateral symmetry (*p* > 0.05; Table 2). In intergroup comparisons, the ranking of the OF diameter followed the order group 3 > group 2 > group 1 (*p* < 0.001; Table 2). A positive correlation was identified between the OF diameter and both body weight and craniometric parameters (*p* < 0.001; Tables S3 and S4). These findings suggest that the size of the OF exhibits a development synchronized with skull size and body mass.

In cattle, the OF area is 67.5 mm^2^ on the right and 71.3 mm^2^ on the left [22]. In our study, the OF area was similar between the right and left body halves in intra-group comparisons (*p* > 0.05; Table 2). However, intergroup comparisons revealed that the OF area increased in parallel with craniometric parameters (*p* < 0.001; Table 2 and Tables S3 and S4). The observed differences in OF sizes among the dog groups may be attributed not only to body size but also to neurovascular requirements. Our study showed that the distance of the OF from the median plane (MOF) in dogs exhibited bilateral symmetry in intra-group comparisons (*p* > 0.05; Table 2). However, in intergroup comparisons, MOF values followed a ranking order of group 3 > group 2 > group 1 (*p* < 0.001; Table 2). Moreover, the positive correlation identified between MOF values and both body weight and craniometric parameters (Tables S3 and S4) demonstrates that this distance exhibits a development synchronized with growth.

In dogs, the SF, through which the middle meningeal artery passes, is defined as a notch (incisura spinosa) or a small foramen rarely located on the aboral and lateral wall of the OF [2,3]. Barone [1] found that this foramen merges with the OF and thus does not appear as a distinct opening. In this study, the position of the SF, identified in 12.5% of the dogs (Figure 5a,b), was consistent with the location of the foramen reported by Nickel et al. [2] and Hermanson et al. [3]. The foramen identified rostral to the OF in two animals (5%) (Figure 5c,d) was consistent with the description provided by Hermanson et al. [3], who noted the same for the middle meningeal artery. In 82.5% (n = 33) of animals, the SF is thought to be fused with the OF, as reported by Barone. A 3D model analysis revealed that the initial segment of the sulcus of the middle meningeal artery on the bones —as reported by Hermanson et al. [3]—was prominent in 18 dogs (10 females and eight males) but was not prominent in 15 dogs (nine females and six males). In one male dog and one female dog, this sulcus (Figure 5c) originated from a bilateral foramen found in the bony septum between the RF and OF. These findings suggest that the presence and location of SF in dogs are related to variations in the middle meningeal artery.

### 4.4. Limitations

The main limitation of this study is the limited sample size due to constraints in material procurement. We could not compare specific breeds due to the lack of full access to the data on specific breeds for all individuals included in the study. Similarly, the effect of age on morphometric parameters could not be analyzed, as a homogeneous distribution of age could not be achieved within the sample group. The extreme scarcity of studies on the cranial base morphology in domestic mammalian species also limits the discussion of our findings from a broader perspective. In this study, instead of the traditional SI based on external skull dimensions, an NL-based approach was preferred to more specifically represent the dimensions of the neurocranium. Although we identified a positive correlation between SI and CI, the mathematical and anatomical power of these two indices is not identical. Therefore, this methodological difference should be considered when directly comparing our data with those of other studies that use traditional SI parameters.

## 5. Conclusions

This study demonstrates that middle cranial fossa foramina maintain bilaterally similar positions and sizes within each dog group, with their dimensions increasing in parallel with skull size, independent of sex, age, and breed. The orbital fissure represents the largest opening in the middle cranial fossa and presents a canal shape in 80% of dogs, while the oval foramen is the second smallest (after the spinous foramen) and exhibits a canal shape in 7.5%. The spinous foramen was observed in 17.5% of the dogs. These findings offer a valuable reference for clinicians, radiologists, and researchers in craniometric studies, skull base surgical planning, regional anesthesia, and the diagnosis of associated neurological disorders. Furthermore, our findings demonstrate that CBCT is highly effective for the cross-sectional analysis of cranial foramina. To ensure more consistent craniometric measurements across varying head shapes and body weights, we propose an NL-based classification approach, the validity of which can be further strengthened by future studies utilizing larger, multi-breed sample groups.

## Figures and Tables

**Table 1:** Descriptive characteristics and craniometric data of 40 dogs. Dogs were divided into three groups according to NL. The statistical results between the groups are presented in the last column.

Mean ± SD (Min. Max.)					
Parameter		Group ^1^ (N = 10)	Group ^2^ (N = 14)	Group ^3^ (N= 16)	*p*
Gender	F	6	5	9	
	M	4	9	7	
Age (year)		5.4 ± 2.83 (2–10)	6.0 ± 4.92 (1–17)	5.15 ± 2.85 (1–11)	
BW (kg)		3.75 ± 1.06 (2.5–6)	15.42 ± 9.74 (7–33)	31.78 ± 8.34 (22–45)	
SL (mm)		97.69 ± 8.40 (81.0–103.5) ^a^	137.55 ± 22.74 (100.83–174.7) ^b^	213.44 ± 22.37 (181.1–258.27) ^c^	<0.001 ***
BL (mm)		84.34 ± 7.86 (68.93–96.53) ^a^	118.90 ± 21.61 (88.43–153.13) ^b^	187.65 ± 17.99 (165.13–226.30) ^c^	<0.001 ***
SW (mm)		70.33 ± 3.05 (64.83–75.50) ^a^	91.41 ± 12.33 (76.47–115.03) ^b^	120.57 ± 14.20 (94.07–145.70) ^c^	<0.001 ***
VL (mm)		40.19 ± 6.90 (29.87–52.57) ^a^	64.05 ± 15.86 (36.60–87.00) ^b^	105.49 ± 12.94 (84.10–125.97) ^c^	<0.001 ***
CL (mm)		63.19 ± 3.74 (57.33–67.53) ^a^	81.98 ± 8.95 (69.37–95.10) ^b^	117.49 ± 11.43 (102.47–143.70) ^c^	<0.001 ***
NL (mm)		60.50 ± 2.81 (56.73–63.67) ^a^	78.32 ± 7.03 (70.10–91.37) ^b^	111.89 ± 8.57 (100.20–131.53) ^c^	<0.001 ***
NW (mm)		48.98 ± 3.52 (45.43–56.30) ^a^	54.28 ± 4.75 (46.56–66.40) ^b^	64.92 ± 4.23 (57.63–73.50) ^c^	<0.001 ***
SI		72.46 ± 6.82 (63.00–83.18) ^a^	67.93 ± 13.85 (55.58–95.36) ^a^	64.92 ± 4.23 (57.63–73.50) ^b^	<0.001 ***
CI		77.61 ± 4.98 (71.22–84.99) ^a^	66.81 ± 8.30 (57.97–85.01) ^b^	55.54 ± 4.40 (48.91–62.39) ^c^	<0.001 ***

Footnotes: BL, basal length; BW, body weight; CI, cranial index; CL, cranial length; F, female; M, male; NL, neurocranium length; NW, neurocranium width; SI, skull index; SL, skull length; SW, skull width; VL, viscerocranium length. *** *p* < 0.001. One-way ANOVA. Different superscript letters within the same row indicate significant differences between groups according to Tukey’s post-hoc test. Groups sharing a common letter are not significantly different. LORF: group ^1^ (n = 3), group ^2^ (n = 13), group ^3^ (n = 16).

**Table 2:** Morphometric data of the right and left foramina in 40 dogs. Dogs were divided into three groups according to NL -. The values in the right and left body halves of each group and the statistical results between the groups are presented in the table.

Parameter (mm, mm^2^)			Mean ± SD (Min. Max.)					
	Side	Group ^1^ (N = 10)	*p* ^1^	Group ^2^ (N= 14)	*p* ^1^	Group ^3^ (N = 16)	*p* ^1^	*p* ^2^
ORF								
LDORF	R	4.69 ± 0.72 (3.60–6.04) ^a^	0.817	5.77 ± 0.50 (4.87–6.95) ^b^	0.275	7.29 ± 0.63 (5.67–8.43) ^c^	0.556	<0.001 ***
	L	4.67 ± 0.70 (3.78–6.11) ^a^		5.65 ± 0.56 (4.47–6.83) ^b^		7.34 ± 0.71 (5.64–8.69) ^c^		<0.001 ***
SDORF	R	2.62 ± 0.25 (2.25–2.94) ^a^	0.155	3.38 ± 0.49 (2.28–4.22) ^b^	0.406	3.99 ± 0.51 (2.82–4.69) ^c^	0.055	<0.001 ***
	L	2.71 ± 0.24 (2.32–3.00) ^a^		3.32 ± 0.52 (2.29–4.29) ^b^		3.85 ± 0.42 (3.18–4.47) ^c^		<0.001 ***
IORF	R	57.14 ± 9.70 (37.32–72.84)	0.217	58.73 ± 8.26 (40.75–75.15)	0.820	54.89 ± 7.10 (38.63–66.02)	0.092	0.444
	L	59.31 ± 10.66 (38.59–79.34)		59.03 ± 9.24 (42.57–80.91)		52.88 ± 7.32 (39.37–67.85)		0.107
LORF	R	2.06 ± 0.05 (2.01–2.12) ^a^	0.184	2.99 ± 0.86 (2.06–4.70) ^a^	0.101	5.44 ± 1.67 (2.02–8.08) ^b^	0.471	<0.001 ***
	L	2.17 ± 0.11 (2.06–2.29) ^a^		2.88 ± 0.89 (2.02–4.68) ^a^		5.29 ± 1.76 (2.34–8.41) ^b^		<0.001 ***
AORF	R	9.66 ± 1.93 (6.08–12.15) ^a^	0.299	13.27 ± 2.32 (10.89–18.60) ^b^	0.983	22.83 ± 3.82 (16.29–28.03) ^c^	0.918	<0.001 ***
	L	9.40 ± 1.75 (6.32–11.68) ^a^		13.27 ± 2.22 (10.63–17.98) ^b^		22.78 ± 3.52 (17.59–27.74) ^c^		<0.001 ***
AnORF (°)	R	23.93 ± 1.21 (22.07–25.73)	0.152	24.79 ± 4.16 (18.83–34.47)	0.124	22.69 ± 1.88 (19.97–27.69)	0.057	0.136
	L	23.76 ± 1.20 (21.90–25.40)		24.94 ± 4.13 (18.93–34.57)		22.54 ± 1.88 (19.77–27.57)		0.077
MORF	R	3.46 ± 0.38 (2.95–4.09)	0.271	4.13 ± 1.50 (2.54–7.36)	0.182	4.63 ± 1.23 (2.80–7.30)	0.260	0.065
	L	3.53 ± 0.42 (3.00–4.27) ^a^		4.23 ± 1.43 (2.60–7.04) ^ab^		4.76 ± 1.16 (2.80–6.88) ^b^		0.038 *
RF								
LDRF	R	3.23 ± 0.54 (2.50–4.09) ^a^	0.533	4.58 ± 0.56 (3.55–5.43) ^b^	0.127	5.78 ± 0.69 (4.63–6.90) ^c^	0.719	<0.001 ***
	L	3.18 ± 0.40 (2.48–3.72) ^a^		4.40 ± 0.66 (3.14–5.90) ^b^		5.87 ± 1.02 (4.12–8.39) ^c^		<0.001 ***
SDRF	R	2.25 ± 0.30 (1.71–2.64) ^a^	0.067	2.94 ± 0.36 (2.21–3.42) ^b^	0.526	3.73 ± 0.40 (2.84–4.67) ^c^	0.929	<0.001 ***
	L	2.14 ± 0.26 (1.69–2.58) ^a^		2.90 ± 0.40 (2.15–3.52) ^b^		3.73 ± 0.38 (2.97–4.19) ^c^		<0.001 ***
IRF	R	70.41 ± 9.49 (60.07–87.22)	0.210	64.87 ± 9.96 (48.14–79.00)	0.346	65.27 ± 9.10 (52.95–76.82)	0.843	0.318
	L	67.58 ± 5.26 (60.88–74.46)		66.68 ± 10.42 (53.93–87.67)		64.75 ± 9.41 (42.89–78.33)		0.710
ARF	R	5.67 ± 1.67 (3.92–8.98) ^a^	0.198	9.33 ± 1.37 (7.12–11.30) ^b^	0.979	15.75 ± 3.11 (10.54–21.73) ^c^	0.747	<0.001 ***
	L	5.38 ± 1.22 (3.84–7.37) ^a^		9.32 ± 1.66 (6.74–11.68) ^b^		15.88 ± 3.30 (10.29–22.71) ^c^		<0.001 ***
MRF	R	5.07 ± 0.43 (4.52–6.00) ^a^	0.211	6.43 ± 0.90 (4.98–7.98) ^b^	0.006**	8.01 ± 1.12 (6.37–9.86) ^c^	0.191	<0.001 ***
	L	5.12 ± 0.46 (4.71–6.17) ^a^		6.51 ± 0.90 (5.01–8.10) ^b^		8.12 ± 1.00 (6.66–10.21) ^c^		<0.001 ***
OF								
LDOF	R	3.49 ± 0.32 (2.90–3.86) ^a^	0.179	4.24 ± 0.74 (3.31–5.49) ^b^	0.719	5.42 ± 0.71 (4.12–6.75) ^c^	0.152	<0.001 ***
	L	3.28 ± 0.49 (2.35–3.88) ^a^		4.26 ± 0.66 (3.47–5.56) ^b^		5.55 ± 0.69 (3.95–6.97) ^c^		<0.001 ***
SDOF	R	1.78 ± 0.28 (1.34–2.27) ^a^	0.496	2.56 ± 0.49 (1.77–3.37) ^b^	0.130	3.40 ± 0.45 (2.63–4.09) ^c^	0.205	<0.001 ***
	L	1.76 ± 0.32 (1.41–2.30) ^a^		2.48 ± 0.43 (1.68–3.15) ^b^		3.21 ± 0.43 (2.45–3.81) ^c^		<0.001 ***
IOF	R	51.04 ± 5.49 (44.19–58.83) ^a^	0.310	60.98 ± 9.29 (42.89–75.24) ^b^	0.081	63.52 ± 10.48 (48.07–91.50) ^b^	0.060	0.005 **
	L	53.73 ± 6.55 (44.36–66.29)		58.71 ± 8.24 (40.55–71.24)		58.14 ± 5.93 (45.79–68.61)		0.194
AOF	R	5.30 ± 1.17 (3.38–7.48) ^a^	0.511	8.43 ± 2.45 (5.20–12.70) ^b^	0.845	14.35 ± 2.90 (9.61–18.35) ^c^	0.140	<0.001 ***
	L	5.17 ± 1.37 (3.47–7.36) ^a^		8.46 ± 2.33 (5.24–12.25) ^b^		14.71 ± 3.15 (10.83–19.14) ^c^		<0.001 ***
MOF	R	8.02 ± 0.45 (7.24–8.63) ^a^	0.130	10.42 ± 0.98 (8.96–12.03) ^b^	0.476	12.44 ± 1.17 (10.93–15.27) ^c^	0.094	<0.001 ***
	L	8.12 ± 0.44 (7.44–8.82) ^a^		10.34 ± 0.97 (8.94–12.20) ^b^		12.67 ± 0.93 (11.10–14.47) ^c^		<0.001 ***

Footnotes: AnORF, angle of orbital fissure; AORF, cross-sectional area of orbital fissure; AOF, cross-sectional area of oval foramen; ARF, cross-sectional area of round foramen; IORF, index of orbital fissure; IOF, index of oval foramen; IRF, index of round foramen; LDORF, longer diameter of orbital fissure; LDOF, longer diameter of oval foramen; LDRF, longer diameter of round foramen; LORF, length of canal of orbital fissure; MORF, distances from ORF to midline; MOF, distances from OF to midline; MRF, distances from RF to midline; OF, oval foramen; ORF, orbital fissure; RF, round foramen; SDORF, shorter diameter of orbital fissure; SDOF, shorter diameter of oval foramen; SDRF, shorter diameter of round foramen. * *p* < 0.05, ** *p* < 0.01, *** *p* < 0.001. *p*^1^= paired sample *t*-test, *p*^2^= one-way ANOVA. Different superscript letters within the same row indicate significant differences between groups according to Tukey’s post-hoc test. Index = shorter diameter/longer diameter × 100. LORF: group ^1^ (n = 3), group ^2^ (n = 13), group ^3^ (n = 16).

## Data Availability

The data that support the findings of this study are available from the corresponding author upon reasonable request.

## Supplementary Materials

The following supporting information can be downloaded at: https://www.mdpi.com/article/10.3390/ani16121819/s1, Figure S1: 3D reconstructive images of skulls from dog groups (lateral view); Figure S2: Craniometric measurements in a female dog. Table S1: Descriptive data of the dogs; Table S2: Intraclass Correlation Coefficient (ICC) values for inter-observer agreement of morphometric measurements in dogs; Table S3: Statistical relationships between age, weight, craniometric data, and parameters related to foramina in groups; Table S4: Statistical relationships between age, weight, craniometric data, and foramen parameters of the three groups; Table S5. Statistical relationships between craniometric data of the three groups.

## Abbreviations

The following abbreviations are used in this manuscript:

BL	Basal Length
BW	Body Weight
CI	Cranial Index
CL	Cranial Length
CT	Computed Tomography
CBCT	Cone-Beam Computed Tomography
DICOM	Digital Imaging and Communication in Medicine
F	Female
FBCT	Fan Beam Computed Tomography
ICC	Intraclass Correlation Coefficient
M	Male
MRI	Magnetic Resonance Images
MPR	Multiplanar Reconstruction
NL	Neurocranium Length
NW	Neurocranium Width
ORF	Orbital Fissure
OF	Oval Foramen
RF	Round Foramen
SF	Spinous Foramen
SI	Skull Index
SL	Skull Length
SW	Skull Width
VL	Viscerocranium Length
3D	Three-Dimensional
