# Supplementary material for: Morphometric Analysis of Foramina in the Middle Cranial Fossa of Dogs: A Retrospective Cone-Beam CT Study
Source: Animals (Basel). 2026 Jun 12;16(12):1819. doi: 10.3390/ani16121819 (PMC13296052; doi:10.3390/ani16121819)
Supplement: Supplementary file 1 [file animals-16-01819-s001.zip › Table S1.pdf]

Table S1. Descriptive data of the dogs.

Animal no	Group	Sex	Age (year)	BW (kg)	NL (mm)	CL (mm)	Breed
1	Group 1	F	2	2.5	56.73	58.63	Chihuahua
2		M	3	4.0	56.93	57.33	Pomeranian
3		F	3	5.0	57.67	59.20	Tibetan spaniel
4		F	9	2.8	58.97	62.93	Chihuahua
5		M	6	3.5	59.63	64.76	Chihuahua
6		M	6	3.2	61.90	66.67	Chihuahua
7		F	10	3.0	62.90	67.53	Pomeranian
8		F	8	6.0	62.93	67.07	Pekingese
9		F	4	3.5	63.63	62.20	Pomeranian
10		M	3	4.0	63.67	65.63	Yorkshire Terrier
11	Group 2	M	2	10.0	70.10	69.37	King Charles Spaniel
12		F	3	7.0	70.80	71.37	Pug
13		M	3	7.5	71.00	74.17	Spitz
14		F	3	7.0	71.67	71.17	Jack Russell Terrier
15		M	1	9.0	73.90	73.53	Jack Russell Terrier
16		M	4	8.0	73.97	79.40	Jack Russell Terrier
17		F	4	11.0	76.17	81.50	French Bulldog
18		M	8	13.0	79.13	90.50	French Bulldog
19		F	3	12.0	79.80	90.33	French Bulldog
20		M	14	N/A	80.60	86.57	N/A
21		M	12	30.0	82.30	82.93	N/A
22		M	8	33.0	87.07	87.80	Golden Retriever
23		M	17	28.0	88.67	94.00	Golden Retriever
24		F	8	25.0	91.37	95.10	Labrador Retriever
25	Group 3	F	7	26.0	100.20	102.47	American staffordshire terrier
26		F	6	45.0	101.47	107.33	Rottweiler
27		F	8	34.0	102.37	103.07	Labrador Retriever
28		F	11	26.0	104.47	108.80	Golden Retriever
29		F	7	30.0	105.03	115.17	Golden Retriever
30		F	1	N/A	105.73	108.17	N/A
31		F	3	N/A	108.83	114.63	N/A
32		M	5	42.0	113.93	114.13	American blue
33		M	5	40.0	113.93	114.67	Cane corsa
34		M	6	26.0	114.00	128.93	Golden Retriever
35		F	9	27.0	114.90	120.87	Labrador Retriever
36		M	1	28.0	115.17	125.20	Pointer
37		M	3	20.0	115.70	112.90	N/A
38		M	3.5	34.0	121.07	127.43	Kangal Shepherd Dog
39		F	5	22.0	121.87	132.37	Border Collie
40		M	2	45.0	131.53	143.70	Kangal Shepherd Dog

N/A: Not available

Abbreviations: *BW, Body weight; CL, Cranial length (inion-nasion); F, Female; M, Male; NL, Neurocranium length (basion-nasion)*
