# Supplementary material for: Morphometric Analysis of Foramina in the Middle Cranial Fossa of Dogs: A Retrospective Cone-Beam CT Study
Source: Animals (Basel). 2026 Jun 12;16(12):1819. doi: 10.3390/ani16121819 (PMC13296052; doi:10.3390/ani16121819)
Supplement: Supplementary file 1 [file animals-16-01819-s001.zip › Table S2.pdf]

Table S2. Intraclass Correlation Coefficient (ICC) values for inter-observer agreement of morphometric measurements in dogs.

Parameter	Side	ICC (95 % CI) Group [1] (n=10)	*p*	Group [2] (n=14)	*p*	Group [3] (n=16)	*p*
**ORF**							
LDORF	R	0.94 (0.84-0.99)	<.001	0.89 (0.73-0.96)	<.001	0.97 (0.92-0.98)	<.001
	L	0.97 (0.89-0.99)	<.001	0.94 (0.85-0.98)	<.001	0.97 (0.92-0.98)	<.001
SDORF	R	**0.71** (0.22-0.92)	0.004	0.93 (0.81-0.98)	<.001	0.96 (0.91-0.99)	<.001
	L	0.88 (0.65-0.97)	<.001	0.97 (0.93-0.98)	<.001	0.96 (0.89-0.98)	<.001
LORF	R	**0.62** (43.31-0.99)	0.226	0.97 (0.93-0.98)	<.001	0.93 (0.98-0.99)	<.001
	L	0.92 (0.18-0.99)	0.029	0.97 (0.93-0.99)	<.001	0.99 (0.97-0.99)	<.001
AORF	R	0.99 (0.96-0.99)	<.001	0.99 (0.98-0.99)	<.001	0.99 (0.97-0.99)	<.001
	L	0.99 (0.96-0.99)	<.001	0.99 (0.98-0.99)	<.001	0.99 (0.97-0.99)	<.001
AnORF (°)	R	0.84 (0.51-0.96)	<.001	0.99 (0.98-0.99)	<.001	0.94 (0.85-0.98)	<.001
	L	0.89 (0.69-0.97)	<.001	0.99 (0.98-0.99)	<.001	0.90 (0.77-0.96)	<.001
MORF	R	0.94 (0.83-0.98)	<.001	0.99 (0.98-0.99)	<.001	0.98 (0.92-0.99)	<.001
	L	0.96 (0.89-0.99)	<.001	0.99 (0.97-0.99)	<.001	0.97 (0.93-0.98)	<.001
**RF**							
LDRF	R	0.94 (0.83-0.98)	<.001	0.86 (0.67-0.95)	<.001	0.86 (0.70-0.95)	<.001
	L	0.92 (0.78-0.98)	<.001	0.94 (0.85-0.98)	<.001	0.96 (0.91-0.98)	<.001
SDRF	R	0.95 (0.85-0.99)	<.001	0.95 (0.87-0.98)	<.001	0.91 (0.80-0.96)	<.001
	L	0.95 (0.85-0.99)	<.001	0.96 (0.89-0.98)	<.001	0.96 (0.91-0.98)	<.001
ARF	R	0.99 (0.96-0.99)	<.001	0.98 (0.96-0.99)	<.001	0.99 (0.98-0.99)	<.001
	L	0.98 (0.95-0.99)	<.001	0.99 (0.97-0.99)	<.001	0.99 (0.98-0.99)	<.001
MRF	R	0.87 (0.63-0.96)	<.001	0.99 (0.97-0.99)	<.001	0.98 (0.96-0.99)	<.001
	L	0.86 (0.57-0.97)	<.001	0.99 (0.97-0.99)	<.001	0.98 (0.96-0.99)	<.001
**OF**							
LDOF	R	0.92 (0.78-0.98)	<.001	0.98 (0.95-0.99)	<.001	0.97 (0.93-0.98)	<.001
	L	0.97 (0.92-0.99)	<.001	0.98 (0.95-0.99)	<.001	0.95 (0.90-0.98)	<.001
SDOF	R	0.97 (0.92-0.99)	<.001	0.96 (0.91-0.99)	<.001	0.95 (0.90-0.98)	<.001
	L	0.96 (0.87-0.99)	<.001	0.97 (0.93-0.98)	<.001	0.95 (0.89-0.98)	<.001
AOF	R	0.99 (0.96-0.99)	<.001	0.99 (0.98-0.99)	<.001	0.99 (0.99-0.99)	<.001
	L	0.98 (0.94-0.99)	<.001	0.99 (0.98-0.99)	<.001	0.99 (0.99-0.99)	<.001
MOF	R	0.96 (0.88-0.99)	<.001	0.98 (0.94-0.99)	<.001	0.95 (0.90-0.98)	<.001
	L	0.96 (0.90-0.99)	<.001	0.97 (0.94-0.99)	<.001	0.92 (0.82-0.97)	<.001

p<0.001, Values in bold indicate a moderate level of agreement between the measurements.

LORF: n=3 for group 1, n=13 for group 2, n=16 for group 3.

ICC; *< 0.50=Poor, 0.50 – 0.75=Moderate, 0.75 – 0.90=Good, > 0.90=Excellent*

Abbreviations: *AnORF, Angle of orbital fissure; AORF, Cross-sectional area of orbital fissure; AOF, Cross-sectional area of oval foramen; ARF, Cross-sectional area of round foramen; LDORF, Longer diameter of orbital fissure; LDOF, Longer diameter of oval foramen; LDRF, Longer diameter of round foramen; LORF, Length of canal of orbital fissure; MORF, Distances from ORF to midline; MOF, Distances from OF to midline; MRF, Distances from RF to midline; OF, Oval foramen; ORF, Orbital fissure; RF, Round foramen; SDORF, Shorter diameter of orbital fissure; SDOF, Shorter diameter of oval foramen; SDRF, Shorter diameter of round foramen.*
