# Supplementary material for: Morphometric Analysis of Foramina in the Middle Cranial Fossa of Dogs: A Retrospective Cone-Beam CT Study
Source: Animals (Basel). 2026 Jun 12;16(12):1819. doi: 10.3390/ani16121819 (PMC13296052; doi:10.3390/ani16121819)
Supplement: Supplementary file 1 [file animals-16-01819-s001.zip › Table S3.pdf]

Table S3. Statistical relationships between age, weight, craniometric data, and parameters related to foramina in groups.

		Group 1									Group 2									Group 3								
		**BW**	**Age**	**SL**	**BL**	**SW**	**VL**	**CL**	**NL**	**NW**	**BW**	**Age**	**SL**	**BL**	**SW**	**VL**	**CL**	**NL**	**NW**	**BW**	**Age**	**SL**	**BL**	**SW**	**VL**	**CL**	**NL**	**NW**
**LDRF**	r	0.429	0.284	-0.050	-0.030	0.480	-0.236	0.187	0.192	0.172	-0.039	0.157	0.129	0.032	0.320	0.012	0.402	0.208	-0.212	-0.212	**,549***	-0.171	-0.153	0.022	-0.018	-0.165	-0.074	0.080
	p	0.217	0.427	0.892	0.935	0.161	0.512	0.605	0.596	0.635	0.898	0.592	0.661	0.912	0.265	0.969	0.154	0.475	0.468	0.467	0.028	0.526	0.572	0.936	0.949	0.540	0.785	0.769
	N	10	10	10	10	10	10	10	10	10	13	14	14	14	14	14	14	14	14	14	16	16	16	16	16	16	16	16
**SDRF**	r	0.589	0.367	0.355	0.368	**,768****	0.097	0.457	0.505	0.197	-0.015	0.161	0.188	0.054	0.468	0.069	0.517	0.330	0.261	0.058	0.154	-0.035	-0.140	0.359	0.064	-0.040	-0.134	0.361
	p	0.073	0.297	0.315	0.296	0.009	0.790	0.184	0.136	0.585	0.962	0.582	0.519	0.854	0.091	0.815	0.058	0.250	0.368	0.843	0.569	0.897	0.604	0.172	0.813	0.882	0.621	0.170
	N	10	10	10	10	10	10	10	10	10	13	14	14	14	14	14	14	14	14	14	16	16	16	16	16	16	16	16
**ARF**	r	**,647***	0.175	-0.049	-0.038	**,822****	-0.285	0.198	0.195	0.216	0.161	0.405	0.360	0.323	0.044	0.290	0.340	0.338	-0.017	0.003	0.153	0.132	0.072	0.222	0.300	0.071	-0.021	0.398
	p	0.043	0.629	0.893	0.917	0.004	0.425	0.584	0.589	0.549	0.600	0.150	0.206	0.260	0.881	0.314	0.235	0.238	0.954	0.993	0.571	0.627	0.791	0.408	0.259	0.794	0.939	0.126
	N	10	10	10	10	10	10	10	10	10	13	14	14	14	14	14	14	14	14	14	16	16	16	16	16	16	16	16
**MRF**	r	0.432	0.613	0.365	0.260	-0.004	0.188	0.478	0.277	-0.144	0.473	0.394	0.198	0.057	**,916*****	0.032	**,720****	**,596***	**,590***	**,581***	-0.389	0.473	0.471	**,749*****	0.398	**,572***	**,679****	**,765*****
	p	0.213	0.060	0.300	0.468	0.991	0.603	0.162	0.439	0.691	0.102	0.164	0.497	0.847	0.000	0.914	0.004	0.024	0.027	0.029	0.136	0.064	0.065	0.001	0.127	0.021	0.004	0.001
	N	10	10	10	10	10	10	10	10	10	13	14	14	14	14	14	14	14	14	14	16	16	16	16	16	16	16	16
**LDOF**	r	0.421	0.209	-0.073	-0.101	**,877*****	-0.331	0.402	0.353	0.597	0.004	0.028	-0.131	-0.291	**,787*****	-0.285	0.510	0.270	**,675****	0.527	-0.328	0.457	0.396	**,790*****	0.380	**,565***	**,594***	**,763*****
	p	0.226	0.562	0.841	0.781	0.001	0.350	0.249	0.318	0.069	0.990	0.924	0.656	0.312	0.001	0.322	0.062	0.351	0.008	0.053	0.215	0.075	0.128	0.000	0.146	0.023	0.015	0.001
	N	10	10	10	10	10	10	10	10	10	13	14	14	14	14	14	14	14	14	14	16	16	16	16	16	16	16	16
**SDOF**	r	0.553	0.226	-0.184	-0.199	**,838****	-0.470	0.257	0.159	0.459	0.364	0.372	0.264	0.098	**,871*****	0.084	**,791*****	**,609***	0.430	**,548***	0.193	0.302	0.215	**,607***	0.258	0.380	0.281	**,618***
	p	0.097	0.531	0.611	0.581	0.002	0.171	0.474	0.660	0.182	0.221	0.190	0.361	0.738	0.000	0.775	0.001	0.021	0.125	0.043	0.473	0.256	0.424	0.013	0.336	0.147	0.291	0.011
	N	10	10	10	10	10	10	10	10	10	13	14	14	14	14	14	14	14	14	14	16	16	16	16	16	16	16	16
**AOF**	r	0.345	0.152	-0.296	-0.330	**,822****	-0.543	0.266	0.120	0.630	0.242	0.252	0.119	-0.043	**,870*****	-0.084	**,724****	0.527	**,628***	0.524	0.033	0.391	0.300	**,724****	0.346	0.468	0.384	**,652****
	p	0.328	0.675	0.406	0.352	0.004	0.105	0.457	0.741	0.051	0.425	0.385	0.685	0.883	0.000	0.775	0.003	0.053	0.016	0.054	0.904	0.134	0.260	0.002	0.189	0.068	0.143	0.006

	N	10	10	10	10	10	10	10	10	10	13	14	14	14	14	14	14	14	14	14	16	16	16	16	16	16	16	16
**MOF**	r	0.432	0.485	**,639***	**,643***	0.052	0.521	0.285	0.340	-0.426	0.459	0.319	0.181	0.076	**,677****	0.094	**,534***	0.491	0.522	**,656***	-0.336	0.375	0.389	**,625****	0.294	0.443	**,523***	**,625****
	p	0.213	0.155	0.047	0.045	0.887	0.122	0.425	0.336	0.219	0.115	0.266	0.536	0.796	0.008	0.750	0.049	0.074	0.056	0.011	0.204	0.152	0.137	0.010	0.269	0.086	0.037	0.010
	N	10	10	10	10	10	10	10	10	10	13	14	14	14	14	14	14	14	14	14	16	16	16	16	16	16	16	16
**LDORF**	r	**,677***	0.140	0.212	0.096	0.389	0.080	0.340	0.138	-0.176	**,598***	0.358	0.460	0.427	0.478	0.375	0.528	**,610***	0.066	0.343	-0.311	0.409	0.335	**,694****	0.338	0.452	0.401	**,663****
	p	0.032	0.699	0.557	0.792	0.266	0.827	0.337	0.703	0.626	0.031	0.209	0.098	0.128	0.084	0.187	0.052	0.021	0.822	0.230	0.241	0.115	0.205	0.003	0.201	0.078	0.124	0.005
	N	10	10	10	10	10	10	10	10	10	13	14	14	14	14	14	14	14	14	14	16	16	16	16	16	16	16	16
**SDORF**	r	-0.317	0.098	-0.098	-0.062	0.175	-0.141	0.068	0.059	0.387	0.525	0.216	0.188	0.144	**,635***	0.047	0.473	0.463	0.231	0.036	-0.391	0.280	0.261	0.283	0.309	0.261	0.278	0.428
	p	0.373	0.787	0.787	0.865	0.629	0.698	0.853	0.871	0.269	0.066	0.459	0.520	0.624	0.015	0.872	0.088	0.096	0.427	0.903	0.135	0.294	0.328	0.289	0.244	0.329	0.297	0.098
	N	10	10	10	10	10	10	10	10	10	13	14	14	14	14	14	14	14	14	14	16	16	16	16	16	16	16	16
**AORF**	r	0.270	0.257	0.268	0.185	0.020	0.158	0.381	0.220	-0.039	0.529	0.182	0.298	0.225	**,648***	0.185	**,565***	**,553***	0.329	0.240	-0.333	0.383	0.325	0.417	0.357	0.378	0.307	**,652****
	p	0.451	0.474	0.454	0.609	0.955	0.663	0.277	0.541	0.914	0.063	0.532	0.300	0.440	0.012	0.528	0.035	0.040	0.251	0.409	0.208	0.143	0.220	0.108	0.174	0.149	0.248	0.006
	N	10	10	10	10	10	10	10	10	10	13	14	14	14	14	14	14	14	14	14	16	16	16	16	16	16	16	16
**LORF**	r	-0.075	0.327	0.481	0.438	-0.375	0.795	-0.994	-0.489	-0.820	**,846*****	**,763****	**,889*****	**,843*****	0.329	**,794****	**,717****	**,895*****	-0.067	0.388	-0.083	0.195	0.092	0.280	0.143	0.247	0.055	0.345
	p	0.952	0.788	0.680	0.712	0.755	0.415	0.071	0.675	0.388	0.001	0.002	0.000	0.000	0.273	0.001	0.006	0.000	0.828	0.170	0.759	0.470	0.734	0.294	0.598	0.356	0.839	0.190
	N	3	3	3	3	3	3	3	3	3	12	13	13	13	13	13	13	13	13	14	16	16	16	16	16	16	16	16
**MORF**	r	0.124	0.425	0.128	0.097	0.343	-0.079	0.468	0.487	0.458	0.121	0.167	-0.189	-0.346	**,854*****	-0.337	0.465	0.225	**,652***	**,613***	-0.067	0.059	0.028	**,895*****	0.019	0.200	0.343	**,593***
	p	0.733	0.221	0.724	0.790	0.331	0.829	0.173	0.153	0.183	0.695	0.569	0.518	0.225	0.000	0.239	0.094	0.439	0.011	0.020	0.807	0.828	0.919	0.000	0.944	0.458	0.193	0.015
	N	10	10	10	10	10	10	10	10	10	13	14	14	14	14	14	14	14	14	14	16	16	16	16	16	16	16	16
**AnORF**	r	-0.396	0.055	-0.532	-0.557	0.115	-0.565	-0.151	-0.394	0.535	-0.216	-0.144	-0.418	**-,561***	**,755****	**-,564***	0.279589	-0.028	**,596***	0.213	-0.216	-0.095	-0.095	-0.276	0.054	-0.117	-0.097	0.296
	p	0.257	0.879	0.114	0.095	0.753	0.089	0.678	0.260	0.111	0.479	0.623	0.136	0.037	0.002	0.036	0.333	0.924	0.025	0.464	0.422	0.726	0.726	0.302	0.843	0.667	0.721	0.266
	N	10	10	10	10	10	10	10	10	10	13	14	14	14	14	14	14	14	14	14	16	16	16	16	16	16	16	16

*p<0.05, **p<0.01,*** p<0.001

Abbreviations: *BL, Basal length (basion-prosthion); BW, Body weight;CL, Cranial length (inion-nasion); F, Female; M, Male; NL, Neurocranium length (basion-nasion); NW, Neurocranium width (euryon-euryon); SL, Skull length (acrocranion-prosthion); SW, Skull width-zygomatic width (zygion-zygion);VL, Viscerocranium length (nasion-prosthion). AnORF, Angle of orbital fissure; AORF, Cross-sectional area of orbital fissure; AOF, Cross-sectional area of oval foramen; ARF, Cross-sectional area of round foramen; LDORF, Longer diameter of orbital fissure; LDOF, Longer diameter of oval foramen; LDRF, Longer diameter of round foramen; LORF, Length of canal of orbital fissure; MORF, Distances from ORF to midline; MOF, Distances from OF to midline; MRF, Distances from RF to midline; SDORF, Shorter diameter of orbital fissure; SDOF, Shorter diameter of oval foramen; SDRF, Shorter diameter of round foramen.*
