# Supplementary material for: Morphometric Analysis of Foramina in the Middle Cranial Fossa of Dogs: A Retrospective Cone-Beam CT Study
Source: Animals (Basel). 2026 Jun 12;16(12):1819. doi: 10.3390/ani16121819 (PMC13296052; doi:10.3390/ani16121819)
Supplement: Supplementary file 1 [file animals-16-01819-s001.zip › Table S4.pdf]

Table S4. Statistical relationships between age, weight, craniometric data, and foramen parameters of the three groups.

		BW	Age	SL	BL	SW	VL	CL	NL	NW
**LDRF**	r	,689***	0.112	,783***	,781***	,789***	,771***	,802***	,820***	,720***
	p	0.000	0.490	0.000	0.000	0.000	0.000	0.000	0.000	0.000
	N	37	40	40	40	40	40	40	40	40
**SDRF**	r	,739***	0.044	,821***	,805***	,861***	,803***	,843***	,841***	,810***
	p	0.000	0.789	0.000	0.000	0.000	0.000	0.000	0.000	0.000
	N	37	40	40	40	40	40	40	40	40
**ARF**	r	,754***	0.036	,852***	,846***	,817***	,848***	,848***	,856***	,812***
	p	0.000	0.827	0.000	0.000	0.000	0.000	0.000	0.000	0.000
	N	37	40	40	40	40	40	40	40	40
**MRF**	r	,847***	0.018	,823***	,805***	,931***	,785***	,881***	,878***	,863***
	p	0.000	0.914	0.000	0.000	0.000	0.000	0.000	0.000	0.000
	N	37	40	40	40	40	40	40	40	40
**LDOF**	r	,762***	-0.091	,789***	,762***	,932***	,740***	,869***	,853***	,903***
	p	0.000	0.575	0.000	0.000	0.000	0.000	0.000	0.000	0.000
	N	37	40	40	40	40	40	40	40	40
**SDOF**	r	,837***	0.110	,832***	,809***	,923***	,792***	,887***	,871***	,854***
	p	0.000	0.498	0.000	0.000	0.000	0.000	0.000	0.000	0.000
	N	37	40	40	40	40	40	40	40	40
**AOF**	r	,825***	0.013	,839***	,815***	,939***	,795***	,897***	,879***	,896***
	p	0.000	0.935	0.000	0.000	0.000	0.000	0.000	0.000	0.000
	N	37	40	40	40	40	40	40	40	40
**MOF**	r	,879***	-0.001	,868***	,859***	,913***	,843***	,896***	,908***	,855***
	p	0.000	0.995	0.000	0.000	0.000	0.000	0.000	0.000	0.000
	N	37	40	40	40	40	40	40	40	40
**LDORF**	r	,835***	-0.025	,878***	,868***	,890***	,852***	,889***	,892***	,810***
	p	0.000	0.878	0.000	0.000	0.000	0.000	0.000	0.000	0.000
	N	37	40	40	40	40	40	40	40	40
**SDORF**	r	,753***	-0.028	,744***	,735***	,795***	,719***	,772***	,777***	,743***
	p	0.000	0.865	0.000	0.000	0.000	0.000	0.000	0.000	0.000
	N	37	40	40	40	40	40	40	40	40
**AORF**	r	,828***	-0.085	,891***	,882***	,876***	,865***	,906***	,908***	,865***
	p	0.000	0.601	0.000	0.000	0.000	0.000	0.000	0.000	0.000
	N	37	40	40	40	40	40	40	40	40
**LORF**	r	,795***	0.150	,760***	,743***	,694***	,740***	,753***	,732***	,667***

	p	0.000	0.414	0.000	0.000	0.000	0.000	0.000	0.000	0.000
	N	29	32	32	32	32	32	32	32	32
**MORF**	r	**,522*****	0.082	**,327***	0.290	**,716*****	0.274	**,458****	**,439****	**,607*****
	p	0.001	0.615	0.039	0.069	0.000	0.087	0.003	0.005	0.000
	N	37	40	40	40	40	40	40	40	40
**AnORF**	r	-0.284	-0.081	**-,339***	**-,379***	-0.053	**-,373***	-0.201	-0.257	0.018
	p	0.089	0.619	0.032	0.016	0.744	0.018	0.214	0.109	0.913
	N	37	40	40	40	40	40	40	40	40

*p<0.05, **p<0.01,*** p<0.001

Abbreviations: *BL, Basal length (basion-prosthion); BW, Body weight; CL, Cranial length (inion-nasion); F, Female; M, Male; NL, Neurocranium length (basion-nasion); NW, Neurocranium width (euryon-euryon); SL, Skull length (acrocranion-prosthion); SW, Skull width-zygomatic width (zygion-zygion);VL, Viscerocranium length (nasion-prosthion). AnORF, Angle of orbital fissure; AORF, Cross-sectional area of orbital fissure; AOF, Cross-sectional area of oval foramen; ARF, Cross-sectional area of round foramen; LDORF, Longer diameter of orbital fissure; LDOF, Longer diameter of oval foramen; LDRF, Longer diameter of round foramen; LORF, Length of canal of orbital fissure; MORF, Distances from ORF to midline; MOF, Distances from OF to midline; MRF, Distances from RF to midline; SDORF, Shorter diameter of orbital fissure; SDOF, Shorter diameter of oval foramen; SDRF, Shorter diameter of round foramen.*
