# Supplementary material for: Morphometric Analysis of Foramina in the Middle Cranial Fossa of Dogs: A Retrospective Cone-Beam CT Study
Source: Animals (Basel). 2026 Jun 12;16(12):1819. doi: 10.3390/ani16121819 (PMC13296052; doi:10.3390/ani16121819)
Supplement: Supplementary file 1 [file animals-16-01819-s001.zip › Table S5.pdf]

**Table S5.** Statistical relationships between craniometric data of the three groups.

| | | SI | CI | SL | BL | NL | CL | VL | SW |
|---|---|---|---|---|---|---|---|---|---|
| **CI** | r | ,704$^{***}$ | | | | | | | |
| | p | 0.000 | | | | | | | |
| | N | 40 | 40 | | | | | | |
| **SL** | r | -,706$^{***}$ | -,911$^{***}$ | | | | | | |
| | p | 0.000 | 0.000 | | | | | | |
| | N | 40 | 40 | 40 | | | | | |
| **BL** | r | -,732$^{***}$ | -,907$^{***}$ | ,996$^{***}$ | | | | | |
| | p | 0.000 | 0.000 | 0.000 | | | | | |
| | N | 40 | 40 | 40 | 40 | | | | |
| **NL** | r | -,593$^{***}$ | -,882$^{***}$ | ,980$^{***}$ | ,978$^{***}$ | | | | |
| | p | 0.000 | 0.000 | 0.000 | 0.000 | | | | |
| | N | 40 | 40 | 40 | 40 | 40 | | | |
| **CL** | r | -,570$^{***}$ | -,886$^{***}$ | ,978$^{***}$ | ,965$^{***}$ | ,988$^{***}$ | | | |
| | p | 0.000 | 0.000 | 0.000 | 0.000 | 0.000 | | | |
| | N | 40 | 40 | 40 | 40 | 40 | 40 | | |
| **VL** | r | -,757$^{***}$ | -,903$^{***}$ | ,991$^{***}$ | ,990$^{***}$ | ,956$^{***}$ | ,949$^{***}$ | | |
| | p | 0.000 | 0.000 | 0.000 | 0.000 | 0.000 | 0.000 | | |
| | N | 40 | 40 | 40 | 40 | 40 | 40 | 40 | |
| **SW** | r | -0.239 | -,749$^{***}$ | ,842$^{***}$ | ,822$^{***}$ | ,898$^{***}$ | ,900$^{***}$ | ,800$^{***}$ | |
| | p | 0.138 | 0.000 | 0.000 | 0.000 | 0.000 | 0.000 | 0.000 | |
| | N | 40 | 40 | 40 | 40 | 40 | 40 | 40 | 40 |
| **NW** | r | -0.266 | -,576$^{***}$ | ,809$^{***}$ | ,788$^{***}$ | ,863$^{***}$ | ,874$^{***}$ | ,771$^{***}$ | ,885$^{***}$ |
| | p | 0.097 | 0.000 | 0.000 | 0.000 | 0.000 | 0.000 | 0.000 | 0.000 |
| | N | 40 | 40 | 40 | 40 | 40 | 40 | 40 | 40 |

*$^{*}p<0.05$, $^{**}p<0.01$, $^{***}p<0.001$*

***Abbreviations:*** *BL, Basal length (basion-prosthion); CI, Cranial index (neurocranium width*100/cranial length); CL, Cranial length (inion-nasion); NL, Neurocranium length (basion-nasion); NW, Neurocranium width (euryon-euryon); SI,Skull index (skull width*100/skull length);*
*SL, Skull length (acrocranion-prosthion);SW, Skull width-zygomatic width (zygion-zygion);*
*VL, Viscerocranium length (nasion-prosthion).*
