# Supplementary figures and images for: Morphometric Analysis of Foramina in the Middle Cranial Fossa of Dogs: A Retrospective Cone-Beam CT Study
Source: Animals (Basel). 2026 Jun 12;16(12):1819. doi: 10.3390/ani16121819 (PMC13296052; doi:10.3390/ani16121819)

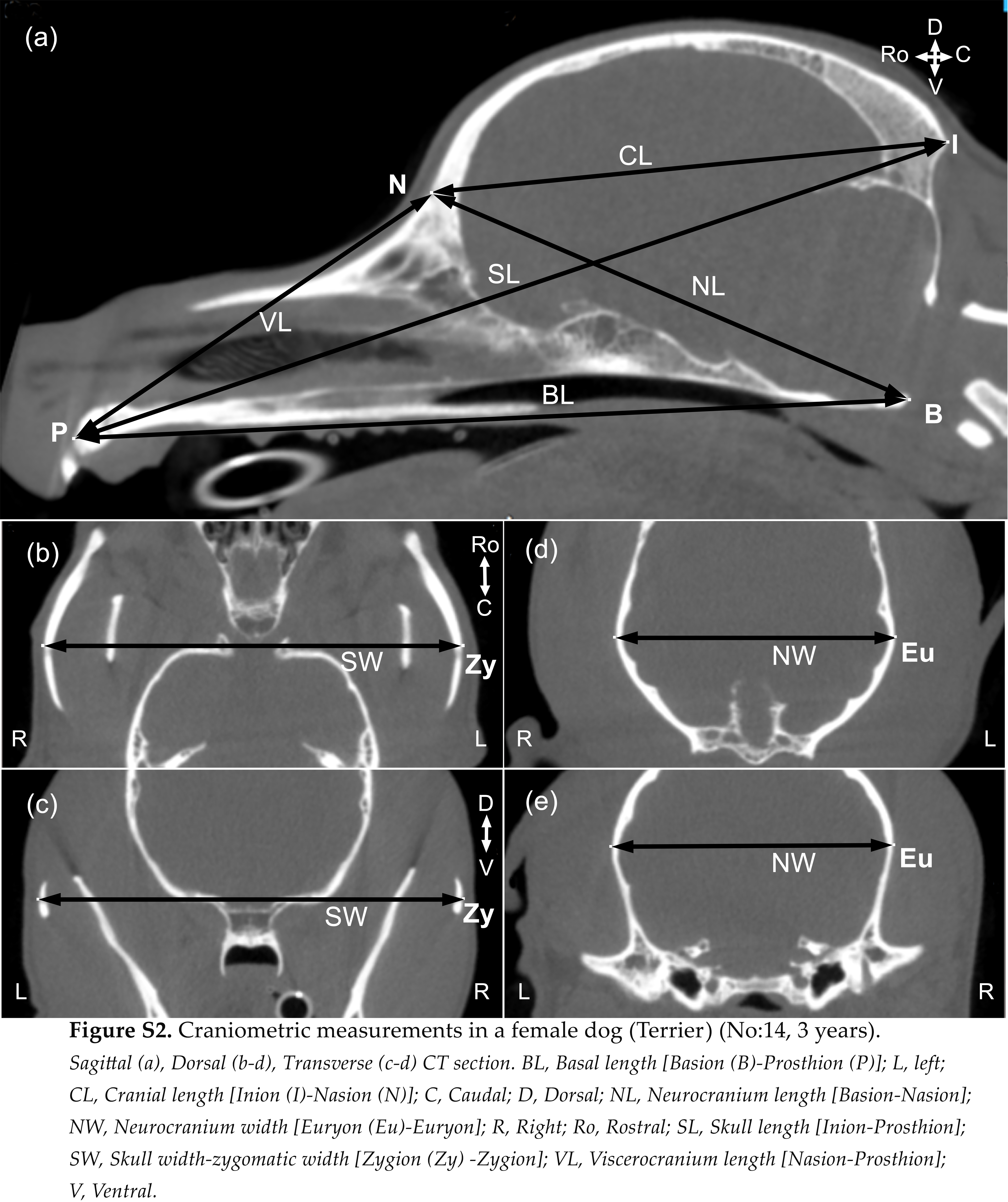

Supplement: Supplementary file 1 [file animals-16-01819-s001.zip › Figure S2.jpg]
